# Truncated Variants of *Gaussia* Luciferase with Tyrosine Linker for Site-Specific Bioconjugate Applications

**DOI:** 10.1038/srep26814

**Published:** 2016-06-08

**Authors:** Eric A. Hunt, Angeliki Moutsiopoulou, Stephanie Ioannou, Katelyn Ahern, Kristen Woodward, Emre Dikici, Sylvia Daunert, Sapna K. Deo

**Affiliations:** 1 University of Miami, Leonard M. Miller School of Medicine, Department of Biochemistry & Molecular Biology, Miami, 33136, USA; 2 University of Miami, Department of Chemistry, Coral Gables, 33146, USA

## Abstract

Gaussia luciferase (Gluc)—with its many favorable traits such as small size, bright emission, and exceptional stability—has become a prominent reporter protein for a wide range of bioluminescence-based detection applications. The ten internal cysteine residues crucial to functional structure formation, however, make expression of high quantities of soluble protein in bacterial systems difficult. In addition to this challenge, the current lack of structural data further complicates the use of Gluc for *in vitro* applications, such as biosensors, or cellular delivery, both of which rely heavily on robust and reproducible bioconjugation techniques. While Gluc is already appreciably small for a luciferase, a reduction in size that still retains significant bioluminescent activity, in conjunction with a more reproducible bioorthogonal method of chemical modification and facile expression in bacteria, would be very beneficial in biosensor design and cellular transport studies. We have developed truncated variants of Gluc, which maintain attractive bioluminescent features, and have characterized their spectral and kinetic properties. These variants were purified in high quantities from a bacterial system. Additionally, a C-terminal linker has been incorporated into these variants that can be used for reliable, specific modification through tyrosine-based bioconjugation techniques, which leave the sensitive network of cysteine residues undisturbed.

In recent years, *Gaussia* luciferase (Gluc)—from the mesopelagic copepod *Gaussia princeps*—has emerged as a prominent reporter protein for bioluminescent detection applications. Gluc exhibits several favorable traits, including high luminescent output, small size (~20 kDa), and excellent stability (including thermostability) in part due to the high number of cysteine disulfides that help form its tertiary structure. The wild-type (WT) sequence for Gluc possesses a 17 amino acid N-terminal excretion tag, and is readily expressed in highly active form in mammalian cell systems^1^,^2^,^3^. As such, Gluc has become a popular reporter for monitoring *in vivo* biological events.

While there is no doubt that Gluc is an excellent bioluminescent reporter for bioanalytical and clinical research applications, there are still some characteristics that make Gluc difficult to work with, and in this regard there have been a number of publications disclosing mutations made to Gluc in order to improve upon or add to its bioluminescent properties. Currently, there is no crystal structure available for Gluc. Therefore, computational techniques and mutagenesis studies must be used to develop conjectures about the regions of the protein that play a significant role in function and substrate interaction.

The sequence for Gluc contains two structural domains exhibiting a high degree of homology (see Fig. 1)^4^. This is a common trait of marine luciferases, and is observed in the *Metridia* luciferase isoforms^5^, which share ~60–70% sequence homology with Gluc. This intramolecular homology is believed to be the result of intragenic duplications and recombinations acting as a mechanism of structural and functional evolution^6^,^7^. Each homologous domain contains five cysteine residues which are intermolecularly conserved among marine luciferases (see Figure S4 of the Supplementary Information). Of these five cysteine residues, four are intramolecularly conserved between the homologous domains (see conserved cysteine residues marked with (*) in Fig. 1). These highly conserved cysteine residues are hypothesized to be involved in the formation of disulfides necessary for tertiary structure formation crucial to functional bioluminescent activity^4^,^8^,^9^,^10^.

In 2009, Maguire *et al.* created a variant of Gluc containing the single point mutation M43I, which provided glow-type kinetics in the presence of 0.1% Triton X-100^11^. This enhanced stability of the bioluminescent emission was favorable for streamlining high-throughput assays utilizing Gluc as a reporter. In 2011, Kim *et al.*^10^ developed several variants of Gluc demonstrating “superluminescent” properties through a semi-rational site-directed approach targeting the hydrophilic region near the end of the second homologous domain. One particular variant—F89W, I90L, H95E, Y97W (Monsta)—demonstrated an approximately five-fold increase in intensity compared to WT Gluc as well as a red-shift in emission to approximately 503 nm^10^. More recently in 2013, Degeling *et al.*^12^ created a variant—L30S, L40P, M43V (Gluc4)—which demonstrated exceptional glow-type kinetics both in the presence and absence of Triton X-100^12^.

These two variants were selected to be further explored in this study. The Monsta variant from Kim *et al.* was chosen for its high bioluminescent output and red-shifted emission. The Gluc4 variant from Degeling *et al.* was was chosen for its exceptional signal stability, however the M43V mutation was replaced with the M43I mutation from Maguire *et al.* Both variants harbor all mutations in the N-terminal homologous domain (see Fig. 1). As such, the N-terminal domain was chosen for creating potential truncated variants with improved bioluminescent activity as well.

In addition to improvements made to the bioluminescent properties of Gluc, there have also been several studies performed to improve the ease by which Gluc is able to be produced. Gluc has traditionally been expressed in mammalian systems as the number of necessary cystine residues make folding in prokaryotic systems difficult. Some solutions to this issue involved expression of Gluc as a fusion with a solubilizing partner such as a synthetic IgG-binding domain^13^, or production of Gluc by cell-free protein synthesis^8^. In 2010, Rathnayaka *et al.*^9^ demonstrated that the use of the pCold expression system (see Figure S5 of the Supplementary Information) enhanced the amount of Gluc present in the soluble fraction when expressed in an LMG194 host^9^. Lowering the temperature at which a protein is expressed slows down the rate of protein production, thereby encouraging proper folding and reducing aggregation^14^.

The difficulty in producing Gluc in high concentration, along with the lack of structural data, limits its use for *in vitro* applications such as biosensors or cellular delivery where reliable bioconjugation is a necessity. Inouye *et al.* introduced a cysteine residue at the end of a linker in an effort to make bioconjugation to Gluc more straightforward^15^. While the use of cysteine residues with traditional maleamide chemistries is well known, it also introduces some extra complications when considering the number of cysteine residues in Gluc involved in disulfide bonds and their sensitivity to reducing conditions which are often necessary to prevent dimerization before conjugation—this is complicated further considering truncation of the sequence, which may expose or otherwise alter the cystine environment.

There are still improvements that can be made to Gluc that will make it easier to work with in a variety of applications and easier to produce in a more affordable and facile expression system, *E. coli*. Expanding on previous work, truncated Gluc variants were created that are characterized by enhanced bioluminescence features, convenient size, and accessible and flexible chemical handles for specific, reliable bioorthogonal conjugation through a tyrosine residue. Large amounts of soluble, properly folded full-length and truncated Gluc variants were expressed and purified from an host without issue utilizing a cold-shock expression vector in conjunction with a knockout strain of bacteria presenting a less reducing environment. The physical manifestations of the mutations present in the Gluc variants were explored using CD spectroscopy, and the resultant kinetic and spectral properties were also determined.

## Results

### Expression and Purification of GlucY Variants from *Escherichia coli*

Gluc requires special attention to expression conditions for proper folding, and while it has been expressed in mammalian systems^1^ it has proven difficult to express in high concentration from bacterial systems. For this reason, the pCold-I expression vector (see Figure S5 of the Supplementary Information) was utilized in conjunction with Origami™ 2 (Novagen - EMD Millipore, Billerica, Massachusetts), a *trxB*^−^/*gor*^−^ strain with K12 background.

The abbreviations for all variants of Gluc used in this study, including inherent mutations, are listed in Table 1. Gluc was purified in high concentration from *E. coli*; 15–20 mg per 1 L culture was recovered from the soluble fraction by immobilized-metal affinity chromatography (IMAC) following cell lysis (see Figure S2). Figure 2 shows the full-length and truncated variants of Gluc produced in this study. The calculated molecular weight for the full-length Gluc variants with C-terminal tyrosine linker is ~22.1 kDa and ~14.4 kDa for the truncated variants. However, the purified proteins appear 4–5 kDa larger when analyzed by SDS-PAGE. This was also observed by Rathnayaka *et al.*^9^.

### Bioluminescence Spectra of GlucY Variants

Spectra were obtained for each Gluc variant using native coelenterazine as the substrate for the bioluminescent reaction. Each full-length variant containing a C-terminal tyrosine linker was compared to the corresponding “native” sequence without the C-terminal linker to ensure that modification of the C-terminus did not disrupt proper folding of the active site. It was determined that the addition of a C-terminal tyrosine linker had no effect on the bioluminescent emission (see Fig. 3A). The truncation of Gluc, however, introduced a 10–15 nm red-shift (see Fig. 3B) in the bioluminescent emission. The emission spectra for each full-length and truncated variant as well as a comparison with *Renilla* luciferase (Rluc) are shown in Fig. 3. The emission maxima for each variant (with tyrosine linker) are as follows: GlucY 480 nm; MonstaY 493 nm; 4lucY 490 nm; tGY 493 nm; tMonY 487 nm; and t4Y 488 nm.

### Kinetics of GlucY Variants

The decay kinetics of the bioluminescent emission of each Gluc variant were determined by adding the native substrate coelenterazine in large excess and recording the relative luminescence until the signal returned to baseline (see Fig. 4). The bioluminescent half-life for each variant determined in this way are listed in Table 2.

In order to compare the relative activity of each variant to the full-length WT sequence, a fixed amount of native substrate coelenterazine was added to a known mass of each variant and allowed to react completely. The emission was integrated in order to calculate quantum yield (*ϕ*), which in this case is a measure of bioluminescence generated per mole of native substrate, coelenterazine. The turnover rate was calculated as a measure of how quickly the substrate was processed in the bioluminescence reaction. As absolute values are sometimes disagreed upon in the literature, both *ϕ* and turnover rate are represented as relative values in comparison to GlucY (see Table 2). This should provide a good representative measure of kinetic parameters as Gluc is a commonly used bioluminescent reporter.

### Secondary Structure Analysis of GlucY Variants by CD Spectroscopy

Circular dichroism (CD) spectra were obtained for all variants containing a tyrosine linker (see Fig. 5A,B) as well as the full-length native variants (see Figure S3 of the Supplementary Information). Secondary structure assignments were made using CDSSTR to analyze the CD spectra (see Fig. 5C–G).

### Modification of Gluc Variants Using FBDP

Specific modification of tyrosine residues with formylbenzene diazonium hexafluorophosphate (FBDP, see Fig. 6A) was conveniently able to be monitored by a change in absorbance in the UV-Visible spectrum centered around 440 nm (see Fig. 6B), and was also confirmed by mass spectrometry (data not shown). GlucY can be successfully modified with 1 equivalent of FBDP without detriment to the bioluminescence.

## Discussion

One of the driving motivations behind the desire to create high-activity truncated variants of Gluc, is that the large size of bioluminescent proteins presents specific challenges in trying to incorporate them as reporters for *in vitro* applications such as biosensors^16^,^17^ or for *in vivo* applications such as cellular delivery for imaging. When conjugating the luciferase to another biomolecule or recognition element, a reduction in size could ultimately equate to a reduction in the likelihood of interference with binding or diffusion of assay components. Most of these applications, especially the production of biosensors, require accurate and reliable bioconjugation techniques to ensure a well characterized product that will function in a reproducible manner. For this reason, in addition to creating truncated variants, a linker peptide containing a reactive tyrosine residue (SLSTPPTPSPSTPPY) was added to the C-terminus of each variant (see Table 1 and Table S3 of the Supplementary Information). This linker was chosen for its proven compatibility with Gluc in both the literature^15^ and other studies performed in our laboratory relating to cysteine modification of Gluc. The tyrosine residue can easily be specifically modified to suit a variety of downstream reactions using diazonium salts^18^,^19^. By using a tyrosine residue for bioconjugation, tight control is maintained over how the GlucY is attached to other biomolecules. This practically eliminates the uncertainty of Gluc orientation—which could hinder bioluminescent function—brought about by using more prominent side chains on the luciferase (e.g. the primary amines of lysine residues). We have successfully used this GlucY to develop a labeled-antibody immunoassay, which will be the topic of another publication.

**Figure 1:**
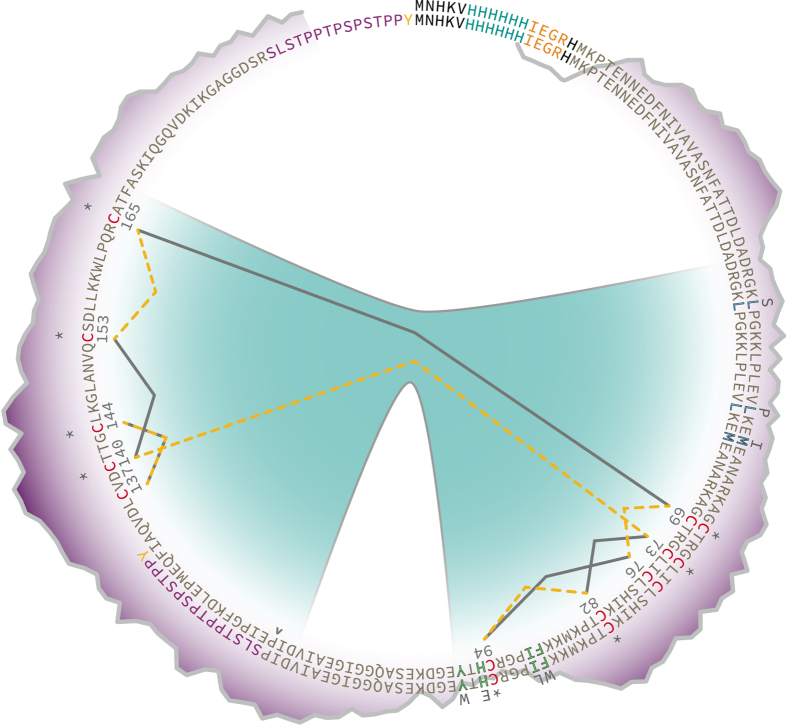
Structural map for GlucY and tGlucY. The two homologous domains of Gluc are indicated by the teal background in the interior of the ring; cysteine residues are colored in red and those conserved between the homologous domains are marked with asterisks (*). Hydrophobicity is plotted around the ring according to the Kyte & Doolittle scale with darker purple being more hydrophobic; the hydrophilic pocket hypothesized to be involved in substrate recruitment is at the bottom of the ring. Cystine predictions are marked with solid grey lines (DiANNA 1.1) and dashed yellow lines (DISULFIND). Though the two algorithms differ slightly in their predictions, both identified two disulfides within each homologous domain. The 4luc mutation points (L47S, L57P, M60I) are shown in outlined blue text and the Monsta mutation points (F89W, I90L, H95E, Y97W) in outlined green text. The mutant residues are listed above each point. The location in the full-length sequence chosen for truncation (E117) is marked by a caret (^).

**Figure 2:**
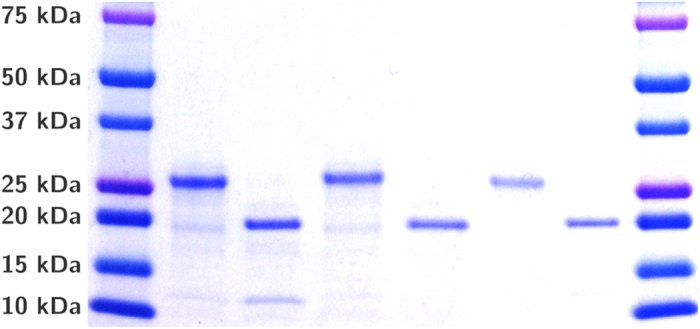
SDS-PAGE analysis of purified Gluc variants with C-terminal tyrosine linker. The first and last lanes contain Precision Plus Protein Dual Color Standard (Bio-Rad Laboratories Inc., Hercules, California). Samples were loaded in the following order: GlucY, tGY, MonstaY, tMonY, 4lucY, t4Y. These diluted crude lysate samples were not treated with protease inhibitor and some degradation is visible.

**Figure 3:**
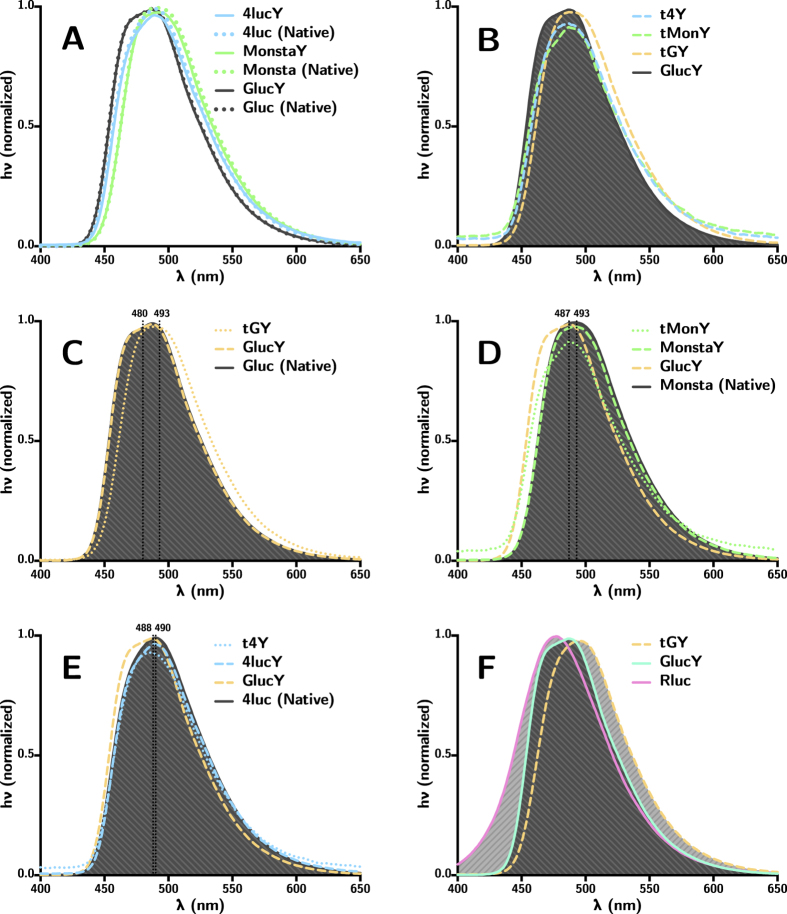
(**A**) The addition of a C-terminal tyrosine linker did not alter the bioluminescence emission of Gluc variants. (**B**) Truncation, however, introduced a 10–15 nm red-shift in the spectrum of each variant. (**C–E**) Comparison of emission spectra for each tyrosine linker variant to its native sequence. (**F**) A comparison to the emission spectra of Rluc is also included. All samples were measured in assay buffer (see Methods section).

The location of cystine disulfides (i.e. which cysteine residues are interconnected) in Gluc has not been determined. As these cystine residues are crucial to the formation of a functional structure, computational techniques^20^,^21^ were used to predict the location of cystine disulfides in Gluc (see Fig. 1). Two internal disulfides were predicted within each homologous domain, with one disulfide bridging the two domains. Curiously, the conserved cysteine residues were not always the ones predicted to be involved in forming the internal disulfides, which would seem logical from an evolutionary standpoint.

Considering the data obtained from CD analysis (see Fig. 5C–G), the addition of a C-terminal tyrosine linker introduced an ~20% increase in unordered structure and corresponding ~20–30% reduction in *α*-helical character (see Fig. 5D), which can be expected as the linker is designed to be a flexible extension for bioconjugation. In general, truncation exhibited the greatest loss in *α*-helical character and corresponding increase in unordered nature for all varaints. Additionally, between the 4luc and Monsta variants, the 4luc mutations introduced the most disorder. This result of these structural changes can be observed kinetically as a reduction in turnover rate, which is likely due to a loss of substrate affinity. Indeed it appears that truncation drastically reduces ability for substrate recruitment.

It appears that the mutations introduced for the MonstaY variant do not cause significant alterations to the secondary structure (see Fig. 5F), with the exception of a slight increase in ordered *α*-helical character. The substrate affinity appears to be enhanced (observed in a greater than three-fold increase in turnover rate), though the relative *ϕ* is reduced—possibly due to consequentially enhanced product inhibition, which is commonly observed among coelenterate luciferases. It is possible that the mutations, while not greatly altering the overall protein structure, provide improved substrate interaction through hydrogen bonding and *π*-interactions, which could potentially explain the red-shift observed for this mutant by increasing electronic interaction between the protein and the substrate.

**Figure 4:**
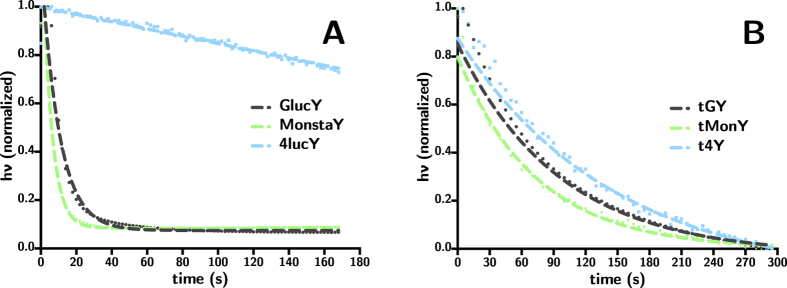
Decay kinetics for (**A**) full-length and (**B**) truncated GlucY variants. All samples were measured in assay buffer (see Methods section).

**Figure 5:**
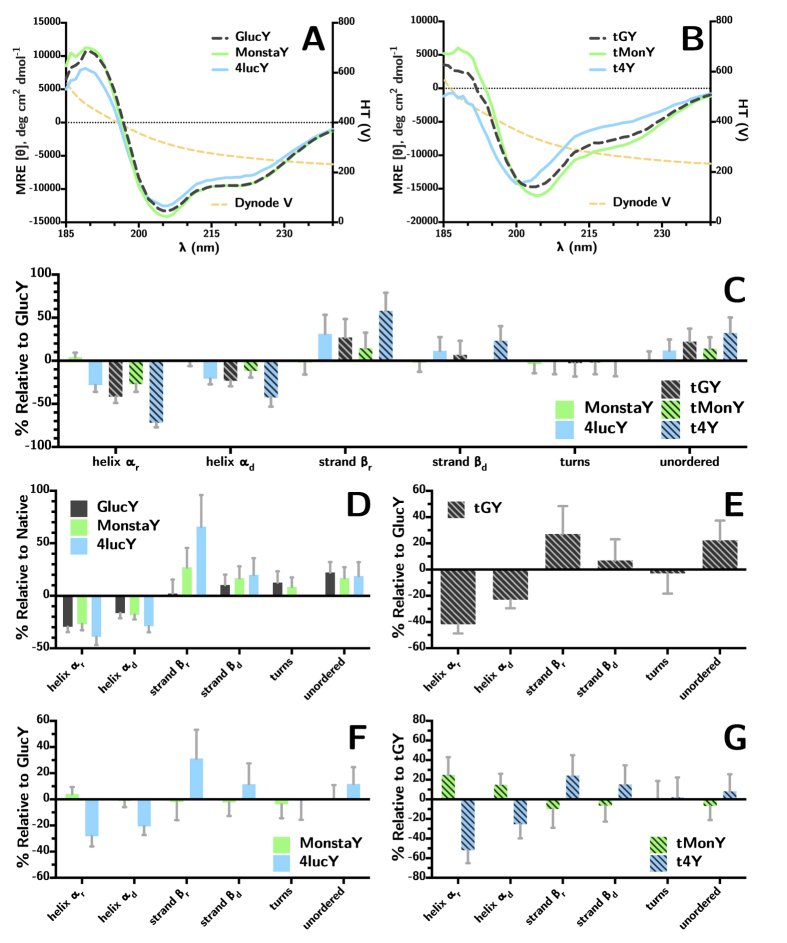
CD spectra for (**A**) full-length GlucY variants and (**B**) truncated GlucY variants. Secondary structure assignments were computed from the CD data using CDSSTR: (**C**) change in all variants relative to GlucY; (**D**) change introduced by C-terminal tyrosine linker relative to native Gluc; (**E**) change introduced by truncation relative to GlucY; (**F**) effect of full-length variant mutations relative to GlucY; (**G**) effect of truncated variant mutations relative to tGY. All samples were measured in CD buffer (see Methods section).

**Figure 6:**
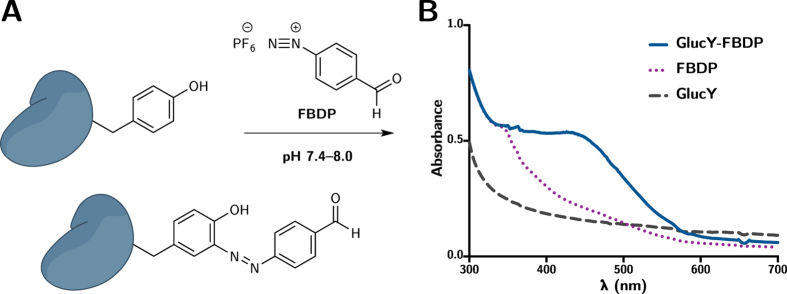
(**A**) Modification of tyrosine residue by FBDP to introduce a bioorthogonal aldehyde for site-specific chemical modification. (**B**) The reaction can be conveniently modified by a change in UV-Visible absorbance. The spectrum was baseline corrected to eliminate any aberration in absorbance that may contribute to the increase observed around 440 nm.

The mutations introduced for the 4luc variant caused more prominent changes to the secondary structure (see Fig. 5F). Kinetically, 4lucY behaves very differently from the other variants, and while its turnover rate is approximately 90% lower, the stability of its emission is exceptional (see Fig. 4A). Indeed, the large change in secondary structure must have drastic effects on its ability to bind the substrate coelenterazine and release the coelenteramide product, as 4lucY shows a four-fold increase in relative bioluminescence *ϕ*.

The wild-type truncated Gluc, tGY, is an attractive truncated luciferase with a similar *ϕ* as the full-size sequence. The reduction in turnover rate corresponds to a more stable luminescent output, making it a potential candidate for high-throughput applications. Unfortunately, though the tMonY and t4Y variants did retain similar spectral properties to their full-length forms, the *ϕ* and turnover rates were greatly reduced (see Table 2). This reduction in *ϕ* and turnover rate for the tMonY and t4Y variants could be due to impaired substrate binding abilities, potentially brought on by the presence of a lone cysteine which may be contributing to alternate, inactive disulfide patterns in conjunction with the other mutations. Further work needs to be done to identify which cysteine residue is involved in the formation of the inter-domain disulfide in the full-length forms (see Fig. 1).

## Conclusion

In conclusion, the successful truncation of the WT sequence and multiple variants of Gluc was carried out. Additionally, a C-terminal tyrosine linker was introduced through molecular cloning. All variants created in this study were characterized by CD spectroscopy and kinetic parameters were derived for comparison of bioluminescent activity. These Gluc variants have wide applicability as bioluminescent reporters given the breadth of their kinetic characteristics and the smaller size of the truncated forms—specifically tGY was the most promising variant in this study, as it is almost half the size of the full-length Gluc sequence and demonstrates a similar bioluminescence *ϕ*. The addition of a tyrosine handle for bioconjugation should prove especially useful in the development of biosensors and targeted cellular delivery systems as well as other classical analytical and biochemical techniques requiring the labeling of other biomolecules or recognition elements with a bioluminescent reporter.

## Methods

### Molecular Cloning

The 185 amino acid sequence for WT Gluc from the NCBI GenBank (accession number AAG54095) was used to create a codon-optimized, synthetic sequence for expression in an *E. coli* host. The signal peptide, which is unnecessary for bacterial expression, was identified using the online prediction tool (see Figure S1 of the Supplementary Information) [http://www.cbs.dtu.dk/] provided by the Center for Biological Sequencing Analysis at the Technical University of Denmark^22^ and removed from the synthetic sequence. The signal peptide region identified has been corroborated by others in the literature^4^.

With the signal peptide removed, the primary sequence for Gluc was codon-optimized for *E. coil* and synthesized commercially (GenScript USA Inc., Piscataway, New Jersey). Two endonuclease restriction sites were included in the synthetic sequence for subsequent cloning steps—a 5′ NdeI restriction site, and a 3′ XbaI restriction site. The commercially synthesized sequence was received cloned into the pUC57 plasmid at the EcoRV restriction site. The pUC57::Gluc and pCold-I (Takara Bio. Inc., Japan) plasmids were transformed into cloning strain NEB5-*α* (New England Biolabs, Ipswich, Massachusetts) for propagation and storage. All plasmid DNA was purified using a QIAprep Spin Miniprep Kit (Qiagen, Valencia, California) before use. The synthetic Gluc sequence was inserted into the pCold-I cold-shock expression vector (see Figure S5 of the Supplementary Information) and verified by Sanger sequencing. Sequencing primers used for the pCold-I vector can be found in Table S2 of the Supplementary Information.

### Mutation and Truncation

Site-directed mutagenesis was performed on pGluc by the polymerase chain reaction (PCR) using the QuikChange Site-Directed Mutagenesis Kit (Agilent Technologies, Santa Clara, California) and the Q5^®^ Site-Directed Mutagenesis Kit (New England Biolabs, Ipswich, Massachusetts) in order to create the variants of Gluc listed in Table 1. A list of primers used for these mutations may be found in Table S2 of the Supplementary Information. PCR primers and methods were developed using the guidelines provided by the online tools PrimerX [http://www.bioinformatics.org/primerx/] and NEBaseChanger [http://nebasechanger.neb.com/]. All mutations were confirmed by Sanger sequencing.

**Figure 7:**
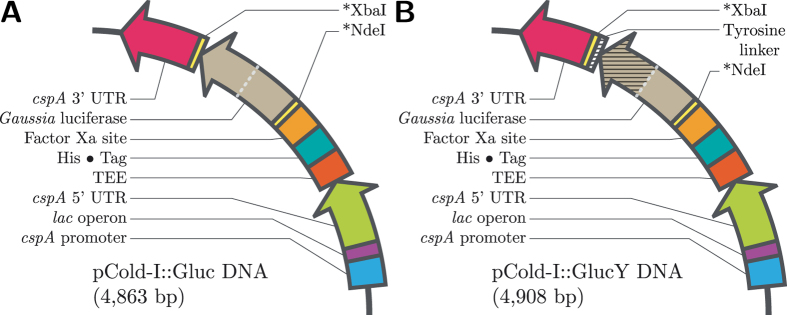
pCold-I plasmid map for Gluc (**B**) with and (**A**) without C-terminal tyrosine linker.

In attempting to create truncated variants of Gluc that retain considerable bioluminescent activity, the location for truncation was chosen with the knowledge that proper cystine disulfide formation is critical to functional structure, and that the hydrophilic region lying between the two homologous domains is important for substrate recruitment. Truncation points were screened by introducing a stop codon into the primary sequence. Once the truncated form was confirmed, a C-terminal tyrosine at the end of a linker peptide sequence was inserted into both the full-length and truncated variants of Gluc using the Q5^®^ Site-Directed Mutagenesis Kit (see Table S2 for primer sequences). For the truncated Gluc, the PCR reaction used to insert the C-terminal tyrosine linker simultaneously deleted the second homologous domain from pGluc (see truncation site marked with (^) in Fig. 1) to eliminate the potential for stop codon read-through. In total, twelve unique plasmids were created for this study (see Table 1 and Fig. 7), six of which contained a C-terminal tyrosine linker.

### Expression and Purification from *Escherichia coli*

The optimized expression protocol was determined to be growth to an OD_600nm_ of 1.6 in Terrific broth supplemented with 0.1 mg/mL ampicillin, replenishment of media following cold-shock on ice for 1 hour, induction with a final concentration of 0.1 mM IPTG, followed by growth at 15 °C overnight. The cells were collected by centrifugation at 4000 × g and 4 °C for 20 minutes and resuspended in a lysis buffer of 50 mM Tris–HCl pH 8.0, 150 mM sodium chloride, 10 mM imidazole, 1% (vol.) nonyl phenoxypolyethoxylethanol (NP-40), 0.2% (vol.) polyoxyethylene (20) sorbitan monolaurate (Tween 20), and 10 mM 2-mercaptoethanol (*β*-ME). The cell suspension was supplemented with 1× ProBlock™ Gold Bacterial Protease Inhibitor Cocktail (Gold Biotechnology Inc., St. Louis, Missouri) and lysed by sonication (Model 500 Sonic Dismembrator, Fisher Scientific, Pittsburgh, Pennsylvania) using a microtip probe for 5–10 minutes (depending on the viscosity of the resuspension of cells in lysis buffer) with a 0.5 second on/off pulse sequence—similar to the method described by Feliu *et al.*^23^. While sonic disruption can potentially decrease protein yield and quality through heat denaturation causing improper folding and the formation of insoluble inclusion bodies, after comparing several methods of lysis, including the use of chemical and pressure differential methods (e.g. BugBusterTM Master Mix [EMD MilliporeTM Corporation, #71456-4] and the use of a French press), this ultrasonic cellular disruption method gave optimal results in yield and protein quality.

The insoluble material was removed by centrifugation at 10,000 × g and 4 °C for 30 minutes and the supernatant was filtered by syringe through a 0.22 μm filter. The filtered crude protein was then incubated with Ni–NTA agarose (Qiagen, Valencia, California) at 4 °C for 45 minutes, collected on a Pierce™ Centrifuge Column (Life Technologies, Grand Island, New York) by gravity flow, washed with 10 column volumes of lysis buffer followed by 20 column volumes of a wash buffer of 50 mM Tris–HCl pH 8.0, 150 mM sodium chloride, and 20 mM imidazole. The protein was then eluted with an elution buffer of 50 mM Tris–HCl pH 8.0, 150 mM sodium chloride, and 150 mM imidazole in 1 column volume increments.

The purified protein was then dialyzed into either an assay buffer of 10 mM sodium phosphate pH 7.2 and 150 mM sodium chloride, or a CD buffer of 5 mM potassium phosphate pH 7.8 and 25 mM ammonium sulfate, depending on the intended application following purification. The described assay buffer was selected for maximum compatibility with other biomolecules and biological samples utilized in our subsequent studies, and the CD buffer was chosen to minimize background during spectroscopic analysis. It is possible that other buffer conditions or components (e.g. the addition of surfactants or ions) could influence the stability and activity of the Gluc variants^4^,^11^. Slide-A-Lyzer™ Dialysis Cassettes (Life Technologies, Grand Island, New York) with a molecular weight cutoff (MWCO) of 10 or 3.5 kDa were used for the full-length and truncated Gluc variants, respectively. The purified protein was typically used within one month, but remained stable without loss of activity in sodium phosphate buffer at 4 °C. Protein concentration was determined using the Bicinchoninic Acid (BCA) Protein Assay Kit (Pierce Biotechnology, Rockford, Illinois) and a SpectraMax 190 Microplate Reader (Molecular Devices, Sunnyvale, California) for absorbance measurements.

### Characterization of Bioluminescence

Native coelenterazine was purchased from NanoLight™ Technology (Prolume Ltd., Pinetop, Arizona). To prepare the coelenterazine for experimental use, 1 mg of lyophilized solid was dissolved in 1 mL of acidified methanol. This stock solution was stored at −80 °C until needed. Working solutions were prepared by diluting the stock solution in PBS.

#### Spectra

Bioluminescence spectra were obtained using a Varian Cary Eclipse Fluorescence Spectrophotometer (Agilent Technologies, Santa Clara, California) equipped with a microplate reader accessory using the bio-/chemi-luminescence mode. All spectra measurements were carried out in 96-well black polystyrene non-binding microplates (Greiner Bio-One Inc., Monroe, North Carolina). Briefly, 100 μL of 300 μM coelenterazine was injected into 200 μL of luciferase varying in concentration from 10 nM to 10 μM—depending on the decay kinetics of each bioluminescence reaction and PMT response—and the spectrum was recorded. Using the Cary Eclipse Scan software package, the spectrum was scanned from 400–650 nm with a 100–500 ms dwell time. Using the software CAT mode, 10 separate spectra were recorded, normalized, and averaged for each reaction.

#### Kinetic Studies

Bioluminescent reaction kinetics were obtained using a POLARstar^®^ Optima (BMG LABTECH GmbH, Ortenberg, Germany). The emission filter was set to “lens” (i.e. no filter) and total light was collected for all measurements. All kinetic measurements were carried out in 96-well black polystyrene non-binding microplates (Greiner Bio-One Inc., Monroe, North Carolina). Briefly, the instrument PMT gain was held constant for all reactions, and the amount of luciferase was varied in order to ensure the emission did not saturate the PMT. The amount of coelenterazine injected to initiate the bioluminescent reaction was held constant at approximately 45 pmol such that the luciferase was always in a ten-fold or greater molar excess—this ensured that the substrate would be processed to completion. Upon injection of coelenterazine, the emission was integrated for 0.1 s at 1 s intervals for 4 min. Excel 2011 (Microsoft Corporation, Redmond, Washington) and the scientific graphing and curve fitting software Prism 6 (GraphPad Software Inc., La Jolla, California) were used to manipulate the raw data and derive the parameters listed in Table 2.

### Circular Dichroism Spectroscopy

CD spectroscopy was performed on a Jasco J-815 Circular Dichroism Spectropolarimeter using a 0.1 cm path length quartz cuvette. Gluc variants were dialyzed into CD buffer and diluted to a concentration of 0.1 mg/mL. Spectra were acquired from 260–185 nm using the following set of instrument parameters: continuous scan mode; 50 nm/min scan speed; 0.5 nm data pitch; 2 nm bandwidth; 2 s integration time; 5 accumulations.

### Computational Analysis of Structure

The two homologous domains present in Gluc were identified using the *Rapid Automatic Detection and Alignment of Repeats* (RADAR) tool [http://www.ebi.ac.uk/Tools/pfa/radar/] hosted by the European Bioinformatics Institute–European Molecular Biology Laboratory (EBI-BMBL) (see Table S1)^6^.

The locations of cystine disulfides in Gluc were predicted using DISULFIND [http://disulfind.dsi.unifi.it/] (Dipartimento di Ingegneria dell’Informazione, Università di Firenze, Firenze, Italy)^20^ and DiANNA 1.1 [http://clavius.bc.edu/clotelab/DiANNA/] (Clote Lab, Boston College, Chestnut Hill, Massachusetts)^21^.

Hydrophobicity data for Gluc was obtained from the ProtScale tool [http://web.expasy.org/protscale/] (ExPASy Swiss Institute of Bioinformatics (SIB) Resource Portal)^24^,^25^ and plotted according to the hydropathy scale developed by Kyte and Doolittle^26^.

All analysis of CD spectra was performed using DichroWeb [http://dichroweb.cryst.bbk.ac.uk/html/home.shtml] (Department of Crystallography, Institute of Structural and Molecular Biology, Birkbeck College, University of London, United Kingdom)^27^. The CDSSTR analysis program^28^ was used to generate secondary structure assignments for Gluc variants in this study using reference sets 3, 4, 6, 7, SP175, and SMP180. The CDSSTR analysis program was chosen as it provided the best fit to experimental data, however, all analysis programs available through DichroWeb including SELCON3, CONTIN, VARSLC, and K2D were evaluated. Data input for analysis was limited to the wavelength range of 240–185 nm.

### Modification of Tyrosine Residues by FBDP

**Figure 8:**
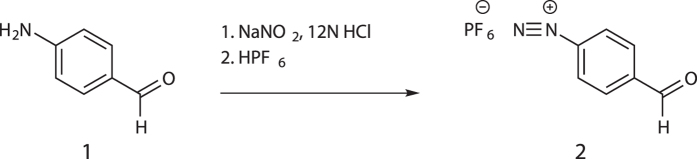
General synthesis of formylbenzene diazonium hexafluorophosphate.

Formylbenzene diazonium hexafluorophosphate (FBDP, **2**) was synthesized according to the protocol described by Gavrilyuk *et al.*^18^ (see Fig. 8). A stock solution of 0.1 M FBDP was prepared in PBS. With a total reaction volume of 1 mL reaction, FBDP was added to Gluc variants with C-terminal tyrosine linker in a 50-fold excess and incubated at room temperature (~20 °C) for 2 h. The modified variants were then buffer exchanged by dialysis (10 kDa MWCO) into a citrate buffer of 100 mM sodium citrate pH 6.0 and 150 mM sodium chloride to remove excess FBDP and prepare the modified variants for subsequent reaction with hydrazine-containing molecules. Specific modification was confirmed by mass spectrometry performed by the Laboratory for Biological Mass Spectrometry at Indiana University (Bloomington, Indiana). FBDP-modified and unmodified samples were submitted to the laboratory for intact and tryptic digest MS analyses. The introduction of an aldehyde functionality by FBDP was also confirmed using an Aldehyde Quantification Assay Kit (Abcam, Cambridge, Massachusetts), and by colorimetric reaction with 2,4-dinitrophenylhydrazine.

## Additional Information

**How to cite this article**: Hunt, E. A. *et al.* Truncated Variants of *Gaussia* Luciferase with Tyrosine Linker for Site-Specific Bioconjugate Applications. *Sci. Rep.* **6**, 26814; doi: 10.1038/srep26814 (2016).

## Supplementary Material

Supplementary Information

## Figures and Tables

**Table 1:** Gluc variants created in this study.

Plasmid	Variant	Mutations	Ref.
pGluc(Y)/ptG(Y)	Gluc(Y)/tG(Y)	*WT (i.e. none)*	–
pMonsta(Y)/ptMon(Y)	Monsta(Y)/tMon(Y)	F89W, I90L, H95E, Y97W	10
p4luc(Y)/pt4(Y)	4luc(Y)/t4(Y)	L47S, L57P, M60I	12,11

The numbering of mutations is from the beginning of the Gluc sequence used in this study and may not match other publications. The M60I mutation differs from the M43V mutation found in the Gluc4 variant^12^ and instead utilizes an M43I mutation^11^. As such, the variant was renamed 4luc for clarity. The plasmids and variants containing the C-terminal tyrosine linker are denoted by a “Y” following their name. The truncated variants and plasmids containing them are denoted by a “t” preceding the variant name.

**Table 2:** Kinetic properties for the bioluminescence reactions of Gluc variants.

	Half-Life (s)	Mass (*μ*g)	Reaction Time* (s)	Relative Quantum Yield (*ϕ*) (% GlucY)	Relative Turnover Rate (% GlucY)
GlucY	7	10 ± 0.25	15	100	100
MonstaY	4	10 ± 0.65	4	62	374
4lucY	†	10 ± 0.95	167	398	9
Tgy	67	10 ± 1.21	104	90	22
tMonY	51	20 ± 0.45	200	5	23
t4Y	101	12 ± 0.34	179	9	16

The quantum yield (*ϕ*) is a measure of luminescence emission per mole of native substrate (coelenterazine). The turnover rate measures how quickly coelenterazine is utilized in the reaction. Both values are expressed relative to GlucY for more straightforward comparison. All samples were measured in assay buffer (see Methods section).

^*^Bioluminescent emission integrated until 90% of maximum was obtained.

^†^4lucY half-life was too long to be determined by regression analysis of the data in Fig. 4A.
