# Supplementary material for: Truncated Variants of Gaussia Luciferase with Tyrosine Linker for Site-Specific Bioconjugate Applications
Source: Sci Rep. 2016 Jun 8;6:26814. doi: 10.1038/srep26814 (PMC4897649; doi:10.1038/srep26814)
Supplement: Supplementary Information [file srep26814-s1.pdf]

Supplementary Information: Truncated Variants of *Gaussia* Luciferase with Tyrosine Linker for Site-Specific Bioconjugate Applications

Eric A. Hunt[1,2], Angeliki Moutsiopoulou[1,2], Stephanie Ioannou[1], Katelyn Ahern[1], Kristen Woodward[1], Emre Dikici[1], and Sapna K. Deo[1,*]

[1]University of Miami, Leonard M. Miller School of Medicine, Department of Biochemistry & Molecular Biology, Miami, 33136, USA
[2]University of Miami, Department of Chemistry, Coral Gables, 33146, USA
[*]sdeo@med.miami.edu

Table S1. Gluc RADAR analysis

No. of Repeats	Total Score	Length	Diagonal	BW-From	BW-To	Level
2	198.08	54	68	43	97	1
43--97	(98.96/50.11)	DRGKLPGKKlPLEVLKEMEANAR.KAGCTRGCLICLSHIKCTPKMKKFIPGRCHTY				
114--168	(99.12/47.08)	DIPEIPGFK.DLEPMEQFIAQVDlCVDCTTGCLKGLANVQCSDLLKKWLPQRCATF				

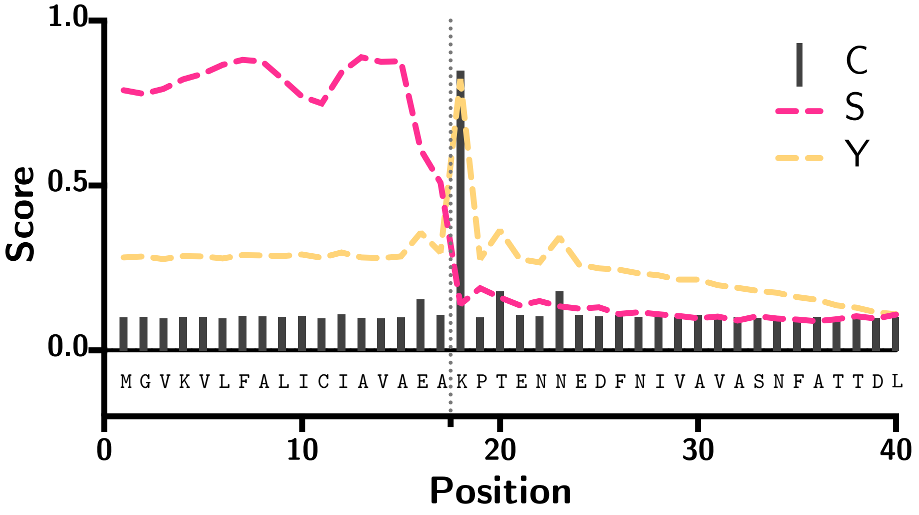

Figure S1. SignalP 4.1 Prediction for Gluc.

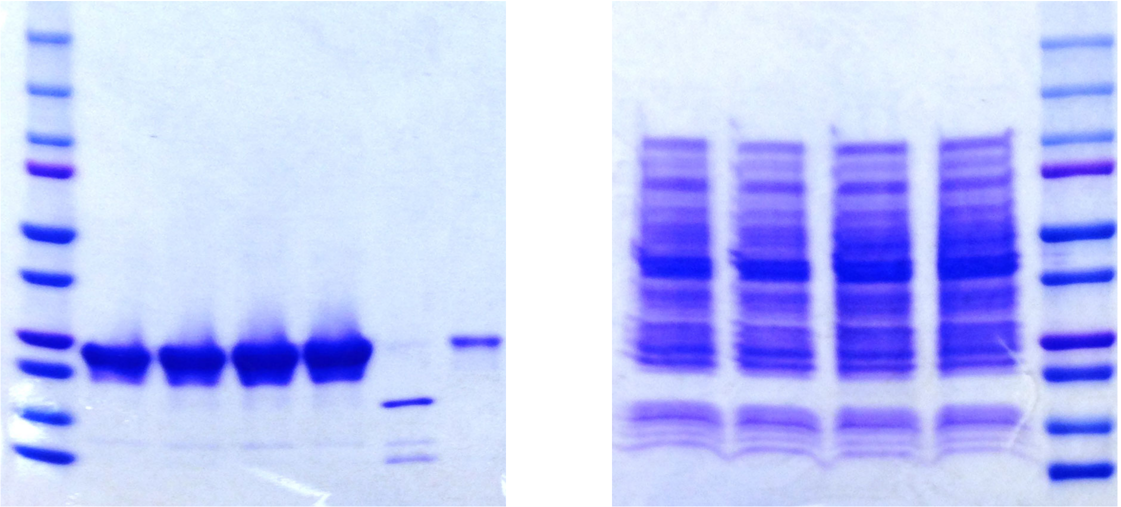

(a) elutions **(b)** flowthrough

Figure S2. Elutions (left) and flowthrough (right) from the optimized expression protocol for Gluc.

Table S2. Primer sequences: the M43I mutation was carried out using the QuikChange kit; the Monsta and 4luc mutations were carried out using the Q5® kit. The codons changed for each mutation are underlined. (Note that the M43I mutation is included in the L40P_f primer and that this mutation differs from the M43V mutation found in Gluc4 produced by Degeling et al.[?]). The tyrosine hinge was introduced using the Q5® kit. The forward primer (luc_f) is the same for both the full-size and truncated reactions. The lowercase portion contains the C-terminal tyrosine insert and is not complementary to pGluc.

Name	(bp)	T_m (°C)	Sequence (printed 5′-->3′)
for Sanger sequencing			
pCold_f	19	69.1	ACGCCATATCGCCGAAAGG
pCold_r	20	31.7	GGCAGGGATCTTAGATTCTG
for Monsta mutations[?] -- F89W/I90L/H95E/Y97W			
F89W/I90L_f	35	85.5	GCCGAAAATGAAAAAATGGCTCCCGGGCCGTTGTC
F89W/I90L_r	40	85.1	GTGCATTTGATATGGCTCAGACAGATCAGGCAACCACGGG
H95E/Y97W_f	41	84.4	GGGCCGTTGTGAGACCTGGGAAGGTGATAAAGAATCTGCAC
H95E/Y97W_r	53	84.3	GGGATAAATTTTTTCATTTTCGGCGTGCATTTGATATGGCTCAGACAGATCAG
for M43I mutation -- M43I			
M43I_f	41	79.6	CTGGAAGTTCTGAAAGAAATTGAAGCAAACGCACGTAAAGC
M43I_r	41	79.6	GCTTTACGTGCGTTTGCTTCAATTTCTTTCAGAACTTCCAG
for 4luc mutations[?] -- L30S/L40P/(*M43I*)			
L30S_f	34	86.6	CTGGACGCTGACCGTGGTAAATCGCCGGGCAAAA
L30S_r	43	83.2	GTCGGTCGTAGCAAAGTTACTCGCCACTGCCACGATGTTAAAG
L40P_f	32	77.8	CTGCCGCTGGAAGTTCCGAAAGAA*ATT*GAAG
L40P_r	27	77.3	TTTTTTGCCCGGCAGTTTACCACGGTC
for tyrosine hinge mutations			
lucY_f	44	56	ctccgtctaccccgccgtactaatTAATCTCTGCTTAAAAGCAC
GlucY_r	43	59	acggggtcggcggggtagacagagaTCTAGAATCACCACCTGC
tGlucY_r	43	58	acggggtcggcggggtagacagagaCGGGATATCGACAATAGC

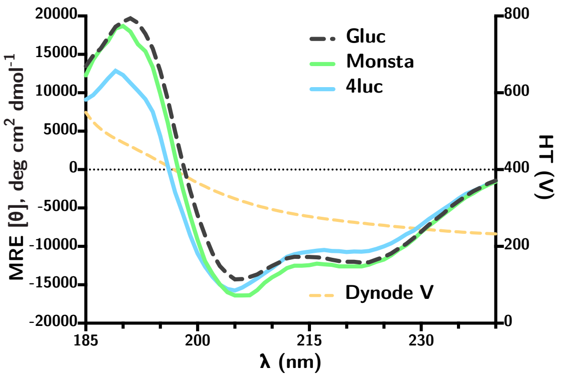

(a) CD spectra for full-size native variants

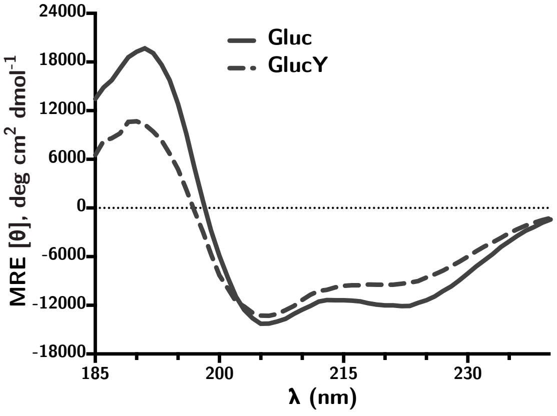

(b) CD spectra for Gluc and GlucY

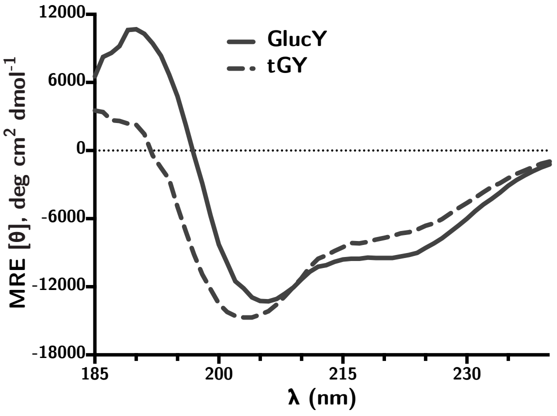

(c) CD spectra for full-size and truncated WT Gluc

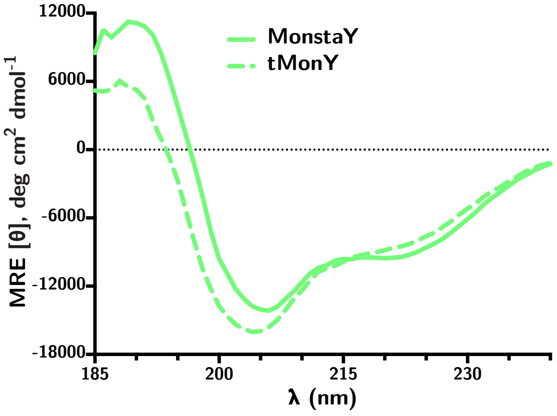

(d) CD spectra for full-size and truncated Monsta

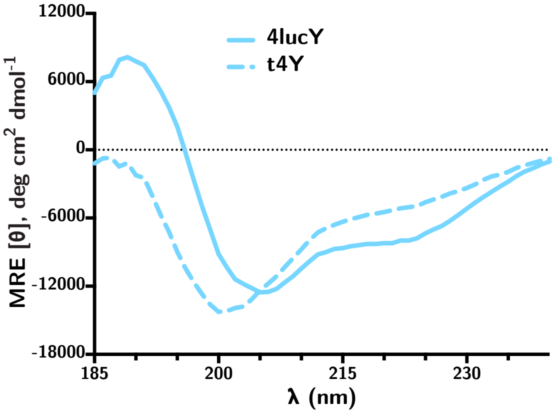

(e) CD spectra for full-size and truncated 4luc

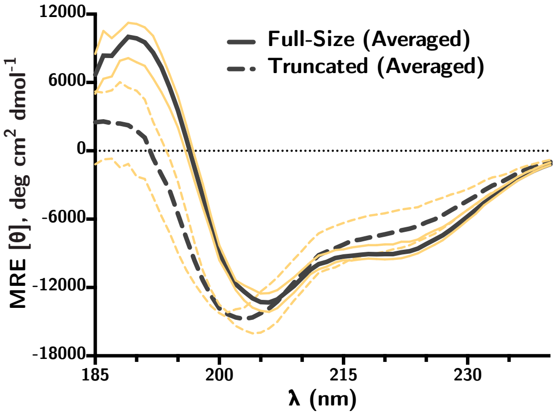

(f) averaged CD spectra for full-size and truncated Gluc variants plotted with range for each dataset

Figure S3. CD comparison spectra overlays

```
Gluc    |AAG54095.1    1  .MGVKVLFALICIAVAEAKP.TENNEDFNIVAVASNFATTDL........
Mluc164 |AAR17541.1    1  .MDIKVVFTLVFSALVQAKS.TEFDPNIDIVGLEGKFGITNLETDLFTIW
Mluc39  |ABW06650.1    1  .MDIKVLFALICIALVQANP.TENNDHINIVGIEGKFGITDLETDLFTIW
Mluc7   |AJC98141.1    1  .MDIKFIFALVCIALVQANP.TVNN.............................
Mpluc1  |BAG48249.1    1  MMEIKVLFALICFALVQANP.TENKDDIDIVGVEGKFGTTDLETDLFTIV
Mpluc2  |BAG48250.1    1  .MGVKLIFAVLCVAAAQAATINENFEDIDVVAIGGSFALD..........

Gluc    |AAG54095.1   41  .........................DADRGK...LPGKKLPLEVLKEMEA
Mluc164 |AAR17541.1   49  ETMEVMIKADIADTDRASNFVATETDANRGK...MPGKKLPLAVIMEMEA
Mluc39  |ABW06650.1   49  ETNRMISTDN..........EQANTDSNRGK...MPGKKLPLAVLIEMEA
Mluc7   |AJC98141.1   24  .........................DVNRGK...MPGKKLPLEVLIEMEA
Mpluc1  |BAG48249.1   50  EDMNVISRDTN..........LVNSDADRGK...MPGKKLPLEVLIEMEA
Mpluc2  |BAG48250.1   40  ........................VDANRGGHGGHPGKKMPKEVLVEMEA

Gluc    |AAG54095.1   63  NARKAGCTRGCLICLSHIKCTPKMKKFIPGRCHTYEGDKESAQGGIGEAI
Mluc164 |AAR17541.1   96  NAFKAGCTRGCLICLSKIKCTAKMKVYIPGRCHDYGGDKKTGQAGIVGAI
Mluc39  |ABW06650.1   86  NAFKAGCTRGCLICLSKIKCTAKMKKYIPGRCHDYGGDKKTGQAGIVGAI
Mluc7   |AJC98141.1   46  NAFKAGCTRGCLICLSKIKCTAKMKQYIPGRCHDYGGDKKTGQAGIVGAI
Mpluc1  |BAG48249.1   87  NARKAGCTRGCLICLSKIKCTAKMKVYIPGRCHDYGGDKKTGQAGIVGAI
Mpluc2  |BAG48250.1   66  NAKRAGCHRGCLICLSHIKCTKKMKKFIPGRCHSYEGDKDSAQGGIGEEI

Gluc    |AAG54095.1  113  VDIPEIPGFKDLEPMEQFIAQVDLCVDCTTGCLKGLANVQCSDLLKKWLP
Mluc164 |AAR17541.1  146  VDIPEISGFKEMAPMEQFIAQVDRCASCTTGCLKGLANVKCSELLKKWLP
Mluc39  |ABW06650.1  136  VDIPDISGFKEMGPMEQFIAQVDRCTDCTTGCLKGLANVKCSELLKKWLP
Mluc7   |AJC98141.1   96  VDIPEISGFKEMEPMEQFIAQVDLCADCTTGCLKGLANVKCSELLKKWLP
Mpluc1  |BAG48249.1  137  VDIPEISGFKELGPMEQFIAQVDLCADCTTGCLKGLANVKCSALLKKWLP
Mpluc2  |BAG48250.1  116  VDMPEIPGFKDKEPMDQFIAQVDLCVDCTTGCLKGLANVHCSALLKKWLP

Gluc    |AAG54095.1  163  QRCATFASKIQGQVDKIKGAGGD.
Mluc164 |AAR17541.1  196  DRCASFADKIQKEVHNIKGMAGDR
Mluc39  |ABW06650.1  186  DRCASFADKIQSEVHNIKGLAGDR
Mluc7   |AJC98141.1  146  DRCASFADKIQKEAHNIKGLAGDR
Mpluc1  |BAG48249.1  187  DRCASFADKIQSEVDNIKGLAGDR
Mpluc2  |BAG48250.1  166  SRCKTFASKIQSQVDTIKGLAGDR
```

Figure S4. Alignment of Gluc with *Metridia* luciferase isoforms. Cysteine residues which are conserved between the internal homologous structural domains are colored red, while those not conserved are colored blue.

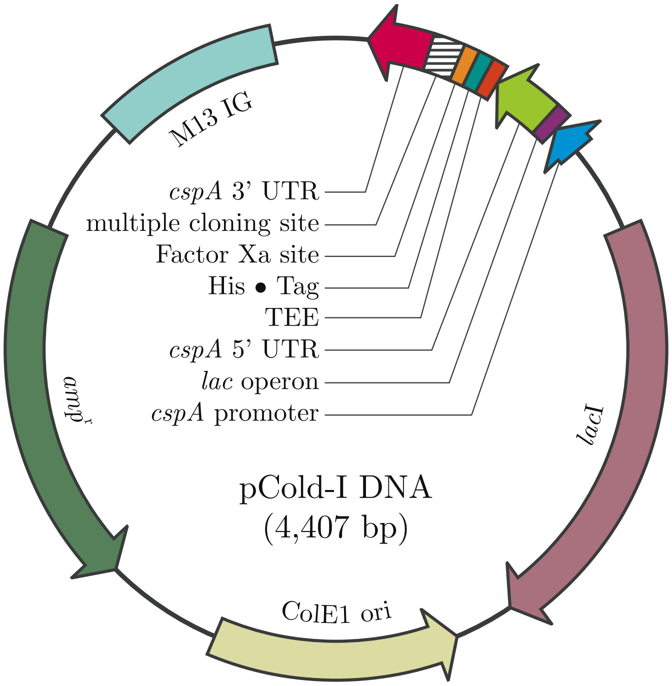

Figure S5. The pCold-I Cold Shock Expression System introduces an N-terminal 6× histidine tag followed by a factor Xa cleavage site for tag removal following purification. The gene of interest inserted into the multiple cloning site is under the control of the *cspA* promoter and *lac* operon for expression control. The vector imparts ampicillin resistance through the *amp*^{r} gene encoding β-lactamase.

Table S3. Primary sequences of Gluc variants with tyrosine linker.

GlucY primary sequence (∧ marks point of truncation; linker is in italics):

```
        10         20         30         40         50         60
MNHKVHHHHH HIEGRHMKPT ENNEDFNIVA VASNFATTDL DADRGKLPGK KLPLEVLKEM

        70         80         90        100        110        120
EANARKAGCT RGCLICLSHI KCTPKMKKFI PGRCHTYEGD KESAQGGIGE AIVDIPEIPG
                                                             ^
       130        140        150        160        170        180
FKDLEPMEQF IAQVDLCVDC TTGCLKGLAN VQCSDLLKKW LPQRCATFAS KIQGQVDKIK

       190        200
GAGGDSRSLS TPPTPSPSTP PY
```

MonstaY primary sequence (∧ marks point of truncation; linker is in italics):

```
        10         20         30         40         50         60
MNHKVHHHHH HIEGRHMKPT ENNEDFNIVA VASNFATTDL DADRGKLPGK KLPLEVLKEM

        70         80         90        100        110        120
EANARKAGCT RGCLICLSHI KCTPKMKKWL PGRCETWEGD KESAQGGIGE AIVDIPEIPG
                                                             ^
       130        140        150        160        170        180
FKDLEPMEQF IAQVDLCVDC TTGCLKGLAN VQCSDLLKKW LPQRCATFAS KIQGQVDKIK

       190        200
GAGGDSRSLS TPPTPSPSTP PY
```

4lucY primary sequence (∧ marks point of truncation; linker is in italics):

```
        10         20         30         40         50         60
MNHKVHHHHH HIEGRHMKPT ENNEDFNIVA VASNFATTDL DADRGKSPGK KLPLEVPKEI

        70         80         90        100        110        120
EANARKAGCT RGCLICLSHI KCTPKMKKFI PGRCHTYEGD KESAQGGIGE AIVDIPEIPG
                                                             ^
       130        140        150        160        170        180
FKDLEPMEQF IAQVDLCVDC TTGCLKGLAN VQCSDLLKKW LPQRCATFAS KIQGQVDKIK

       190        200
GAGGDSRSLS TPPTPSPSTP PY
```

Linker (italics in original): *SLS TPPTPSPSTP PY*
